# The role of nuclear factor erythroid-2-related factor 2 expression in radiocontrast-induced nephropathy

**DOI:** 10.1038/s41598-019-39534-2

**Published:** 2019-02-22

**Authors:** Ji Eun Kim, So Yeon Bae, Shin Young Ahn, Young Joo Kwon, Gang Jee Ko

**Affiliations:** 1 Department of Internal Medicine, Korea University College of Medicine, Seoul, Korea; 2 Nephrology Research Institution, Korea University Guro Hospital, Seoul, Korea

## Abstract

Radiocontrast-induced nephropathy (CIN) is the third most common cause of acute renal failure. The pathophysiology of CIN is related to tubular injury caused by oxidative stress, and nuclear factor erythroid-2-related factor 2 (Nrf2) is critical in coordinating intracellular antioxidative processes. We thus investigated the role of Nrf2 in CIN. CIN was established in mice and in NRK-52E cells via iohexol administration according to the protocols of previous studies. To determine the role of Nrf2 in CIN, Nrf2 expression was reduced *in vivo* using Nrf2 knockout (KO) mice (B6.129 × 1-Nfe2 l2tm1Ywk/J) and *in vitro* with siRNA treatment targeting Nrf2. Increased Nrf2 expression was observed after iohexol treatment both *in vivo* and *in vitro*. Serum creatinine at 24 h after iohexol injection was significantly higher in KO mice than in wild-type (WT) mice. Histologic examination showed that iohexol-induced tubular vacuolization and structural disruption were aggravated in Nrf2 KO mice. Significant increases in apoptosis and F4/80(+) inflammatory cell infiltration were demonstrated in KO mice compared to WT mice. In addition, the increase in reactive oxygen species after iohexol treatment was augmented by Nrf2 inhibition both *in vivo* and *in vitro*. Nrf2 may be implicated in the pathogenesis of CIN via the modulation of antioxidant, anti-apoptotic, and anti-inflammatory processes.

## Introduction

Contrast-induced nephropathy (CIN) is the third most common cause of acute kidney injury (AKI). The incidence of CIN has increased in recent years, owing in part to the increased frequency of examinations and invasive contrast-based procedures and to the increased number of patients with chronic kidney disease who are susceptible to CIN^1,2^. A number of studies have suggested that radiocontrast agents generate reactive oxygen species (ROS), which can cause ischemic tubular injury and direct tubular toxicity^1,3,4^. In other studies, ROS scavengers, such as N-acetylcysteine, have shown a protective effect against CIN by reducing ROS production^5–8^. However, the detailed mechanisms of the development of CIN remain unclear.

Nuclear factor erythroid-2-related factor 2 (Nrf2) is a key factor in the coordination of the intracellular antioxidative process^9–11^. Nrf2 is stimulated under conditions of increased cytoplasmic oxidative stress, such as hypoxia or chemical stress^12,13^, and has shown a protective role against renal ischemic-reperfusion injury in murine models, as well as a role in reducing renal and possibly even systemic inflammation among hemodialysis patients^14–16^. Here, we used *in vitro* and *in vivo* murine models to evaluate the role of Nrf2 as a therapeutic target of CIN.

## Results

### Nrf2 is associated with iohexol-induced renal injury

**Figure 1:**
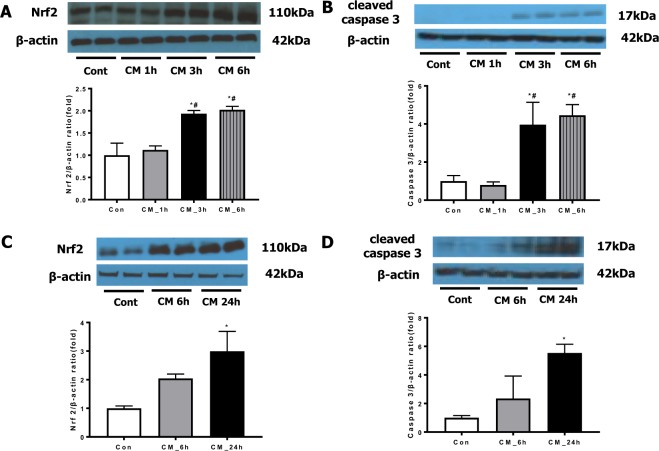
Effects of radiocontrast on nuclear factor, erythroid 2 like 2 (Nrf2) and cleaved caspase 3 expression. Representative western blot analyses of Nrf2 and cleaved caspase 3 expression over time after iohexol treatment and quantitative analyses are shown: *in vitro* (**A**,**B**) and *in vivo* (**C**,**D**) experiments. The expression of Nrf2 was significantly increased after iohexol treatment. Cropped gels are used in the figure, and the full-size gels are presented in Supplementary Figures S5–8. *p < 0.05 *vs*. Cont and ^#^p < 0.05 *vs*. CM 1 h. Abbreviations: Cont, saline control group; CM, iohexol treatment group.

**Figure 2:**
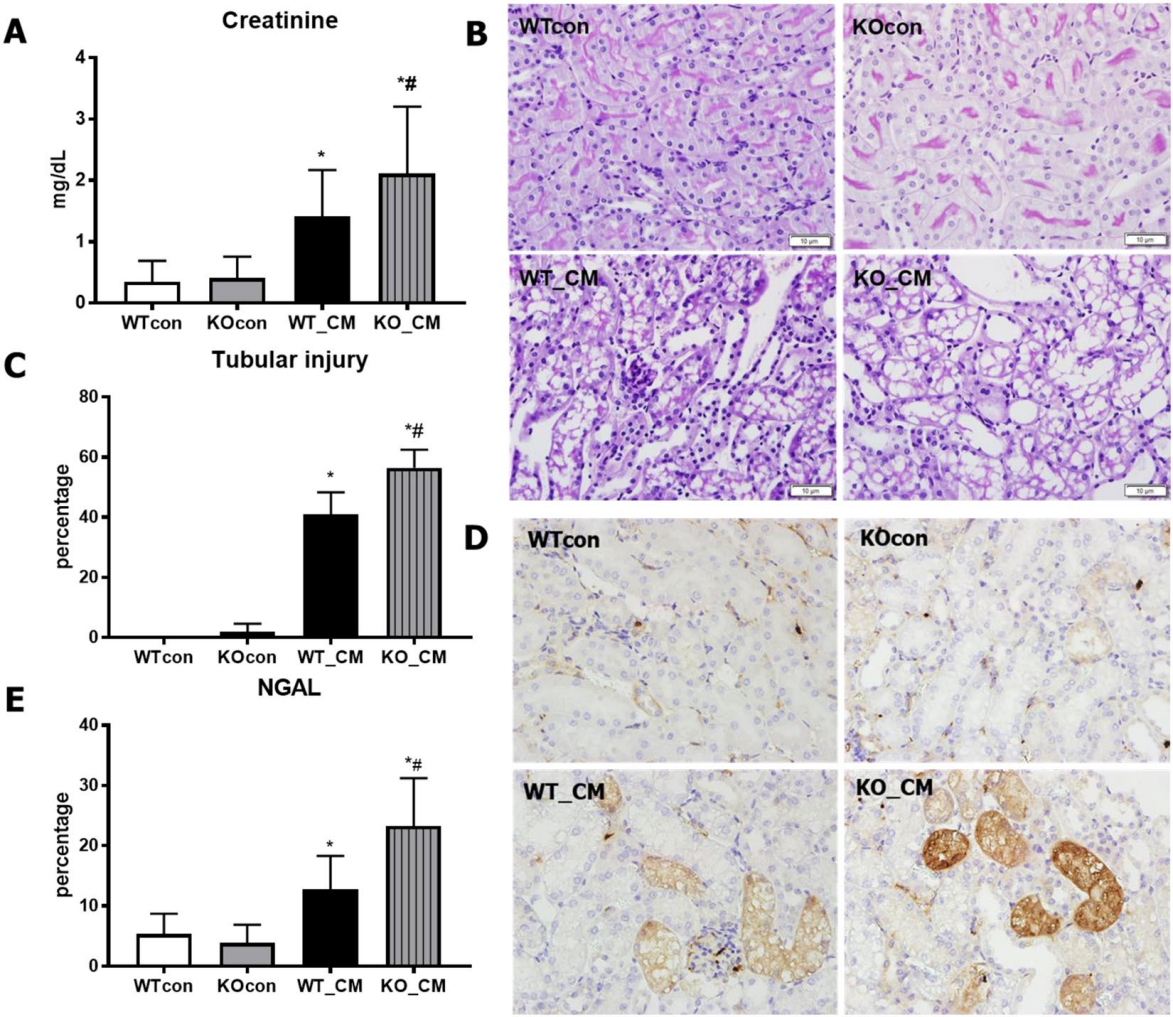
Changes in creatinine levels, histologic findings in the kidneys and renal injury markers after iohexol treatment. (**A**) Increased serum creatinine levels after iohexol treatment were aggravated by the loss of Nrf2 function. *p < 0.05 *vs*. WTcon and KOcon, ^#^*p* < 0.05 *vs*. WT_CM. (**B**) Representative histology of the tubular changes after contrast injury. Iohexol treatment in mice induced tubular dilatation and vacuolization with tubular structure disruption. The severity and extent of tubular injury were aggravated upon the loss of Nrf2 (periodic acid-Schiff (PAS) staining; original magnification, ×400). (**C**) Quantitative measurement of tubular injury in histology by measuring the area of tubular dilatation and vacuolization. The area of tubular injury after iohexol treatment was significantly increased in the group with Nrf2 loss compared to that of the other groups. *p < 0.05 vs. WTcon and KOcon, ^#^*p* < 0.05 *vs*. WT_CM. (**D**) Immunohistochemical staining of NGAL. Iohexol treatment in mice increased NGAL staining area and intensity in tubules, and these effects were further aggravated by the loss of Nrf2 (NGAL; Original magnification, ×400). (**E**) The area of NGAL positive staining was calculated and compared between groups. The histology of iohexol-treated Nrf2 KO mice showed significantly increased NGAL staining in renal tubules compared to that of the other groups. *p < 0.05 vs. WTcon and KOcon, ^#^p < 0.05 *vs*. WT_CM. Abbreviations: WTcon, wild-type control group; KOcon, Nrf2 knockout control group; WT_CM, wild-type iohexol-treated group; KO_CM, Nrf2 knockout iohexol-treated group; Nrf2, nuclear factor, erythroid 2 like 2.

After iohexol treatment, Nrf2 expression was increased over time both *in vivo* and *in vitro*, and it was correlated with the degree of injury, as assessed by cleaved caspase-3 expression (Fig. 1). WT mice treated with iohexol had significantly higher serum creatinine levels than the untreated WT control mice (WT_CM *vs*. WTcon: 1.41 ± 0.76 *vs*. 0.35 ± 0.34 mg/dL, *p* < 0.05). Although the loss of Nrf2 function itself did not alter creatinine levels (KOcon: 0.41 ± 0.34 mg/dL, *p* > 0.05 compared to WTcon), iohexol-treated Nrf2-KO mice had significantly higher creatinine levels than iohexol-treated WT mice (KO_CM: 2.12 ± 1.09 mg/dL, *p* < 0.05 compared to WT_CM; Fig. 2A). Histological evaluation of kidney sections by PAS staining revealed significant tubular vacuolization with the disruption of tubular structures in the cortex and outer stripe of the medulla in WT mice treated with iohexol, and the loss of Nrf2 exacerbated the degree and area of tubular injury (Fig. 2B). The calculated tubular injury value was significantly higher in iohexol-treated Nrf2-KO mice than in iohexol-treated WT mice and controls (Fig. 2C). Additionally, the immunohistochemistry examination of NGAL, a marker for tubular injury, showed that renal damage measured by the NGAL-positive area was significantly higher in iohexol-treated Nrf2-KO mice than in the other groups (Fig. 2D,E).

### Knockdown of Nrf2 reduces cell viability after iohexol treatment

**Figure 3:**
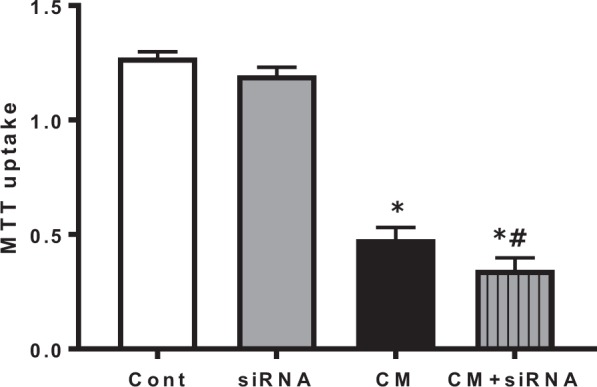
Cell cytotoxicity assays after iohexol treatment and Nrf2 inhibition. Nrf2 inhibition aggravated the decrease in cell viability measured by MTT assay after iohexol treatment. *p < 0.05 *vs*. Cont and siRNA, ^#^*p* < *0*.*05 vs*. CM. Abbreviations: Cont, control group; siRNA, siRNA transfection group; CM, iohexol-only treatment group, CM + siRNA, iohexol-treated group with Nrf2 inhibition by siRNA transfection; Nrf2, nuclear factor, erythroid 2 like 2; MTT, 3-(4,5-dimethylthiazol-2-yl)-2,5-diphenyltetrazolium bromide.

Gene silencing of Nrf2 via siRNA transfection was performed in NRK-52E cells, and quantitative polymerase chain reaction (qPCR) was performed to evaluate the efficacy of Nrf2 knockdown. Compared to scrambled control siRNA treatment, specific siRNA transfection significantly suppressed Nrf2 expression (Supplementary Fig. S1). Cell viability measured by MTT assay was significantly reduced after iohexol treatment (Cont *vs*. CM: 1.273 ± 0.075 *vs*. 0.479 ± 0.268, *p* < 0.05). Although cell viability was not affected by scrambled siRNA transfection itself (siRNA: 1.196 ± 0.108, *p* > 0.05 compared to Cont), iohexol treatment after inhibiting Nrf2 via siRNA transfection significantly decreased cell viability compared to iohexol-only treatment (CM + siRNA: 0.346 ± 0.269, *p* < 0.05 compared to CM; Fig. 3).

### Loss of Nrf2 promotes iohexol-induced apoptosis *in vivo* and *in vitro*

**Figure 4:**
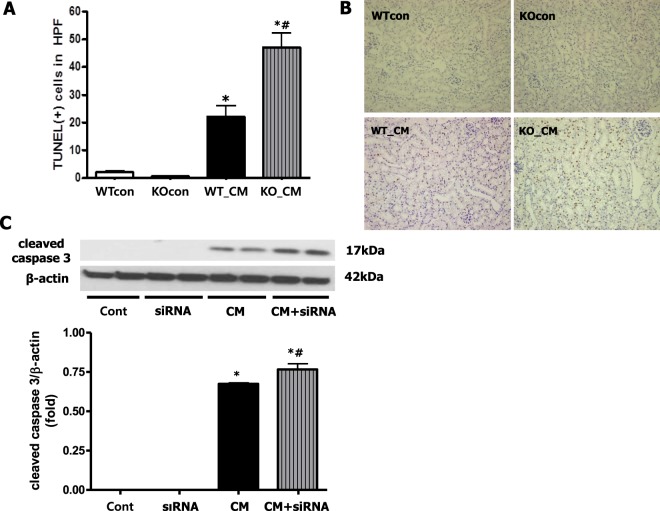
Loss of Nrf2 in mice enhanced iohexol-induced apoptosis. Terminal deoxynucleotidyl transferase deoxyuridine triphosphate nick-end labeling (TUNEL) staining of kidney tissue (**A**) and quantification of TUNEL-positive apoptotic nuclei (B) revealed an increase in apoptosis in renal tubules after iohexol treatment. In addition, this effect was aggravated by the loss of Nrf2. *p < 0.05 vs. WTcon and KOcon, ^#^*p* < 0.05 vs. WT_CM. (**C**) Representative immunoblot analysis and quantitative analysis showed increased cleaved caspase 3 expression in tubular cells after iohexol treatment. Inhibition of Nrf2 enhanced cleaved caspase 3 expression. Cropped gels are used in the figure, and the full-size gels are presented in Supplementary Figure S9. *p < 0.05 *vs*. Cont and siRNA, ^#^*p* < *0*.*05 vs*. CM. Abbreviations: WTcon, wild-type control group; KOcon, Nrf2 knockout control group; WT_CM, wild-type iohexol-treated group; KO_CM, Nrf2 knockout iohexol-treated group; Cont, control group; siRNA, siRNA transfection group; CM, iohexol-only treatment group, CM + siRNA, iohexol treated group with Nrf2 inhibition by siRNA transfection; Nrf2, nuclear factor, erythroid 2 like 2.

The degree of apoptosis was assessed by TUNEL staining in an *in vivo* experiment, and the number of TUNEL-positive apoptotic cells was significantly increased after iohexol treatment (WTcon, KOcon, and WT_CM: 2.0 ± 1.6, 0.7 ± 0.7, and 22.1 ± 16.5/HPF, respectively, *p* < 0.05 compared to WTcon and KOcon). The loss of Nrf2 aggravated the increase in apoptotic cells in renal tubules following iohexol treatment (KO_CM: 47.0 ± 22.0/HPF, *p* < 0.001 compared to WT_CM; Fig. 4A,B). Cleaved caspase-3 expression was evaluated to monitor apoptosis *in vitro* using renal tubular cells, and Nrf2 inhibition significantly enhanced the increase in cleaved caspase-3 expression after iohexol treatment (Fig. 4C).

### Loss of Nrf2 enhances iohexol-induced oxidative stress and ROS production

**Figure 5:**
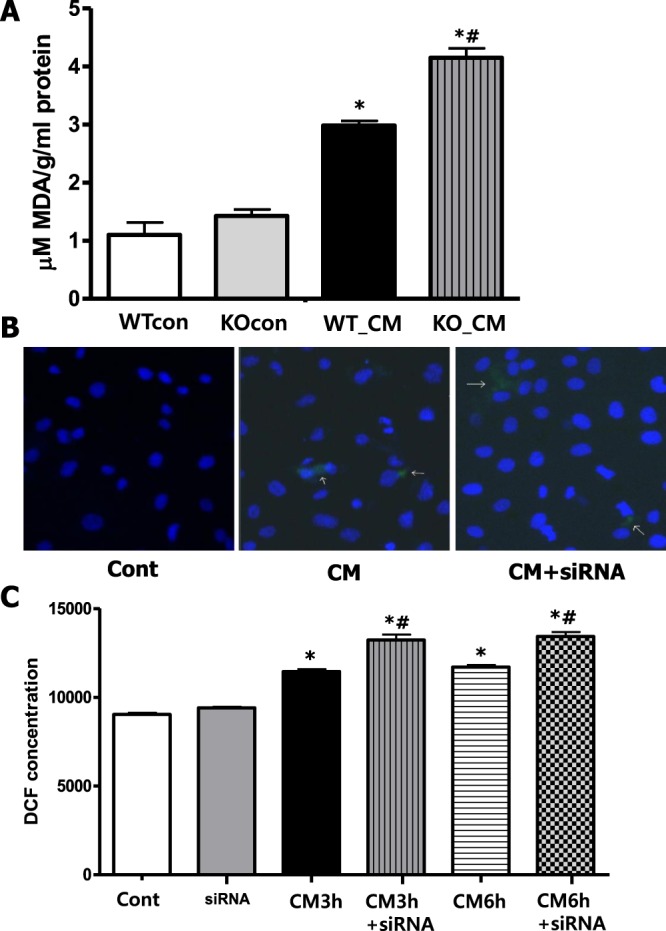
Loss of Nrf2 enhanced the increase in iohexol-induced oxidative stress. (**A**) Oxidative stress in the kidneys determined by MDA concentration was increased after iohexol treatment, and this effect was augmented in mice with the loss of Nrf2 function. *p < 0.05 *vs*. WTcon and KOcon, ^#^*p* < 0.05 *vs*. WT_CM. (**B**) ROS production according to DCF-DA staining was observed by confocal microscopy (2000× magnification). Green-colored immunofluorescence was increased after iohexol treatment, and this effect was enhanced by Nrf2 inhibition. (**C**) The percentage of fluorescence intensity of DCF-stained cells measured by a fluorescence spectrophotometer showed similar results. *p < 0.05 *vs*. Cont and siRNA, ^#^*p* < *0*.*05 vs*. CM 3 h and 6 h. Abbreviations: WTcon, wild-type control group; KOcon, Nrf2 knockout control group; WT_CM, wild-type iohexol-treated group; KO_CM, Nrf2 knockout iohexol-treated group; Cont, control group; siRNA, siRNA transfection group; CM, iohexol-only treatment group, CM + siRNA, iohexol-treated group with Nrf2 inhibition by siRNA transfection; Nrf2, nuclear factor, erythroid 2 like 2; DCF-DA, dichlorofluorescein diacetate.

Next, we measured the intensity of oxidative stress after iohexol treatment in mice by MDA analysis. Iohexol-treated mice exhibited increased oxidative stress (WTcon, KOcon, and WT_CM: 1.101 ± 0.370, 1.428 ± 0.192, and 2.990 ± 0.134 mM MDA/g/mL protein, respectively, *p* < 0.05 compared to WTcon and KOcon), and the loss of Nrf2 resulted in a significant increase in oxidative stress in the kidneys (KO_CM: 4.152 ± 0.280, *p* < 0.05 compared to WT_CM; Fig. 5A). Upon measuring ROS directly in renal tubular cells using DCF-DA, increased ROS production was detected after iohexol treatment according to immunofluorescence microscopic examination. ROS levels were further enhanced by Nrf2 inhibition (Fig. 5B). The quantification of ROS production by spectrophotometry revealed similar results, as ROS production was increased after iohexol treatment, independent of the duration of exposure, and was further augmented by Nrf2 inhibition (Fig. 5C).

### Loss of Nrf2 decreases HO-1 expression and increases cytochrome c expression *in vivo* and *in vitro*

**Figure 6:**
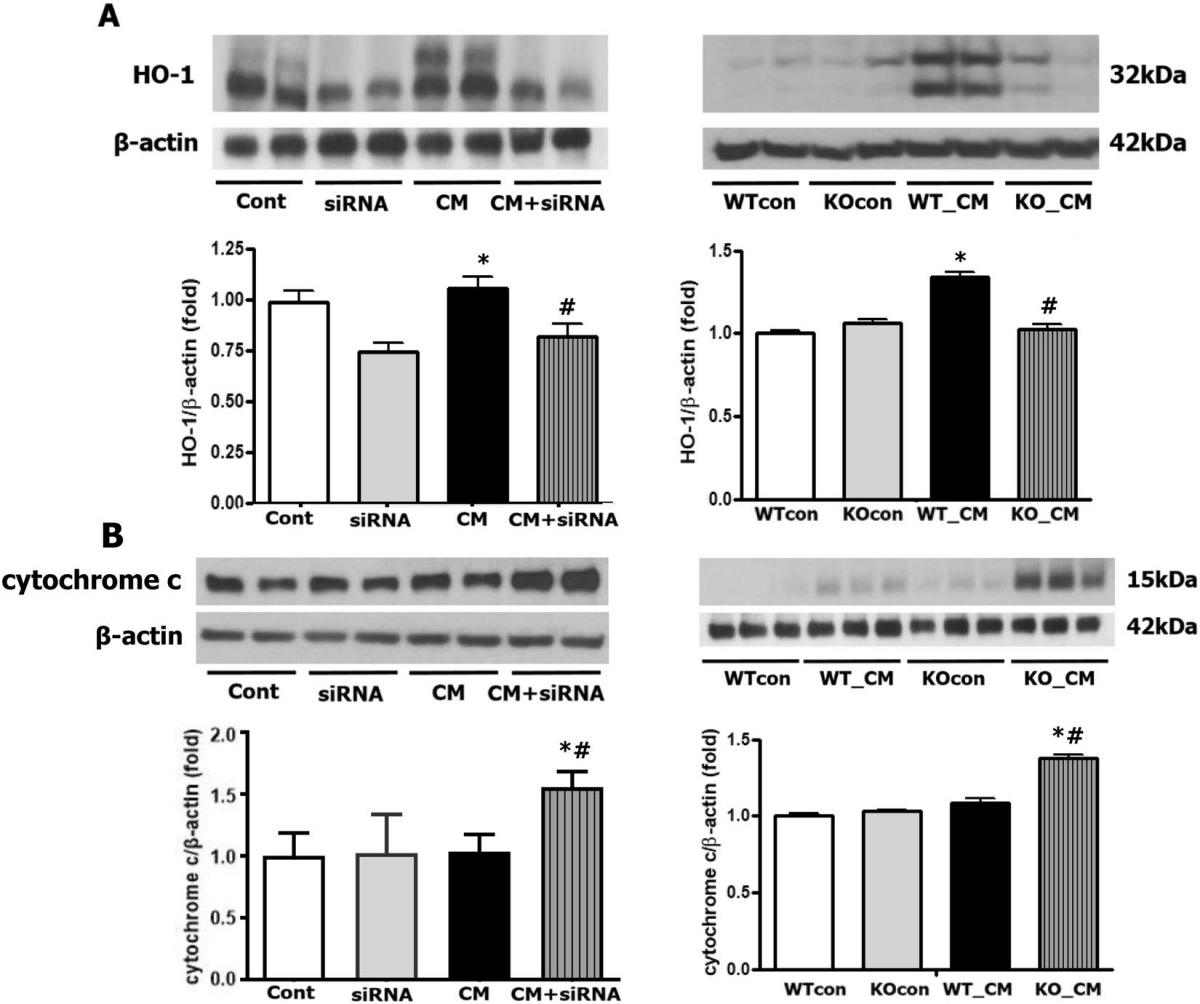
Immunoblot analyses of HO-1 and cytochrome *c*. An increase in HO-1 expression after iohexol treatment was not observed upon reduced Nrf2 function in both *in vivo* and *in vitro* experiments (**A**). Reduced Nrf2 function was also associated with enhanced cytochrome *c* expression. Cropped gels are used in the figure, and the full-size gels are presented in Supplementary Figure S10. (**B**). *p < 0.05 *vs*. Cont/siRNA or WTcon/KOcon. ^#^p < 0.05 vs. CM or WT_CM. Cropped gels are used in the figure, and the full-size gels are presented in Supplementary Figure S11. Abbreviations: Cont, control group; siRNA, siRNA transfection group; CM, iohexol-only treated group, CM + siRNA, iohexol-treated group with Nrf2 inhibition by siRNA transfection; WTcon, wild-type control group; KOcon, Nrf2 knockout control group; WT_CM, wild-type iohexol-treated group; KO_CM, Nrf2 knockout iohexol-treated group; Nrf2, nuclear factor, erythroid 2 like 2.

Heme oxygenase-1 (HO-1) is a well-known regulatory enzyme involved in oxidative stress that mediates Nrf2-associated antioxidant signaling. HO-1 was not clearly expressed in the saline-treated WTcon or KOcon group, while its expression was markedly increased in iohexol-treated WT mice. However, the loss of Nrf2 blocked HO-1 induction in Nrf2-KO mice after iohexol treatment. Similarly, Nrf2 inhibition in tubular cells reduced HO-1 activation after iohexol treatment (Fig. 6A). Reduced Nrf2 function was also associated with enhanced cytochrome c expression (Fig. 6B).

### Loss of Nrf2 stimulates iohexol-induced F4/80(+) macrophage infiltration into tubules

**Figure 7:**
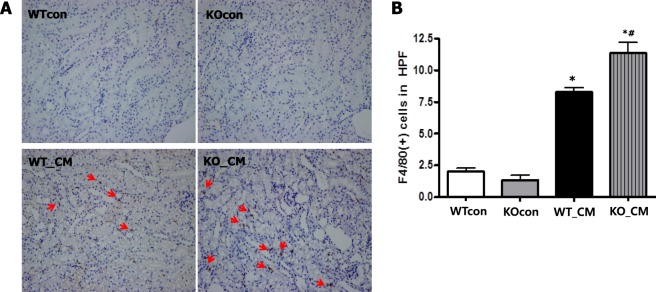
Loss of Nrf2 promotes macrophage infiltration in the renal tubules after iohexol treatment. Immunohistochemical staining of F4/80 (**A**) and quantitative analysis of F4/80 positive cells (red arrow, (**B**) showed increased macrophage infiltration after iohexol treatment, and these effects were augmented by the loss of Nrf2 function. *p < 0.05 *vs*. WTcon and KOcon, ^#^p < 0.05 *vs*. WT_CM. Abbreviations: WTcon, wild-type control group; KOcon, Nrf2 knockout control group; WT_CM, wild-type iohexol-treated group; KO_CM, Nrf2 knockout iohexol-treated group; Nrf2, nuclear factor, erythroid 2 like 2.

Macrophage infiltration was examined by F4/80(+) immunohistochemistry. F4/80(+) macrophage infiltration into mouse kidneys was significantly increased at 24 h after iohexol treatment (WTcon and KOcon *vs*. WT_CM: 2.0 ± 1.0 and 1.3 ± 1.3 vs. 8.3 ± 1.9/HPF, respectively, *p* < 0.001 compared to WTcon and KOcon). Moreover, the loss of Nrf2 enhanced F4/80(+) macrophage infiltration into renal tubules after iohexol treatment (KO_CM: 11.4 ± 3.9/HPF, *p* < 0.001 compared to WT_CM; Fig. 7).

### Nrf2 activation with CDDO-Me treatment attenuates iohexol-induced tubular cell injury

**Figure 8:**
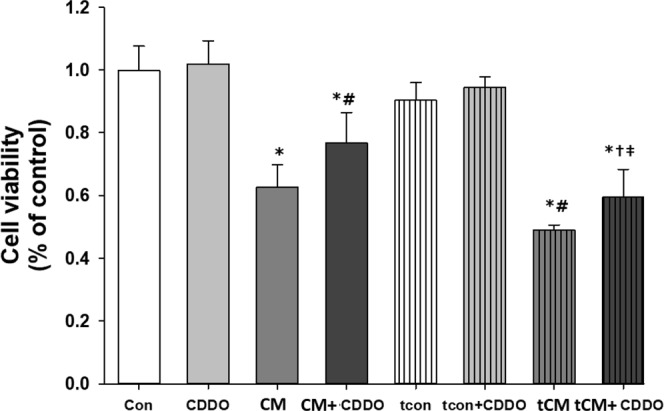
Quantitative analysis of cell viability by 5-(3-carboxymethoxyphenyl)-2H-tetrazolium inner salt (MTS) assay after iohexol and Nrf2 activator (CDDO-Me) co-treatment with or without the inhibition of Nrf2 expression by siRNA transfection in NRK-52E cells. Cell viability was not changed upon pretreatment of CDDO itself. However, decreased cell viability after iohexol treatment was significantly attenuated by Nrf2 activation with 6-hour CDDO pretreatment. Nrf2 inhibition by siRNA pretreatment enhanced the decrease in cell viability after iohexol treatment, and CDDO pretreatment partially recovered the cell viability decrease induced by iohexol and Nrf2 inhibition ^*^p < 0.05 *vs*. Con, CDDO, tcon, and tcon + CDDO, ^#^p < 0.05 *vs*. CM, and ^†^p < 0.05 *vs*. CM + CDDO, ^‡^p < 0.05 *vs*. tCM.

CDDO-Me was used as an Nrf2 activator, and its effect on Nrf2 expression was examined by qPCR. CDDO-Me treatment increased Nrf2 gene expression significantly compared to control treatment (p = 0.05, Supplementary Figure S2). Treatment with the Nrf2 activator CDDO-Me itself did not alter cell viability in NRK-52E renal tubular cells according to the MTS assay results (Cont *vs*. CDDO: 1.000 ± 0.068 *vs*. 0.996 ± 0.096 *p* > 0.05). However, CDDO-Me and iohexol co-treatment significantly attenuated the iohexol-induced decrease in cell viability (CM *vs*. CM + CDDO: 0.647 ± 0.090 *vs*. 0.736 ± 0.089, *p* < 0.05). Nrf2 inhibition exacerbated the decrease in cell viability after iohexol treatment (tCM: 0.490 ± 0.031, *p* < 0.05 compared to Cont and CDDO), and this effect was partially attenuated by CDDO-Me co-treatment (tCM + CDDO: 0.539 ± 0.084, *p* < 0.05 compared to tCM; Fig. 8).

## Discussion

In this study, we demonstrated that Nrf2, a transcription factor acting as a key regulator of various cellular defense enzymes, was closely associated with the pathophysiology of CIN. Nrf2 expression in the kidney was increased after contrast media (CM) administration, and the inhibition of Nrf2 expression exacerbated CIN while increasing tubular cell apoptosis and ROS production. Reduced expression of the antioxidant enzyme HO-1 with the functional loss of Nrf2 and the subsequent increase in intracellular stress are possible underlying mechanisms. Attenuation of CIN upon co-treatment with the Nrf2 activator CDDO-Me supported the protective role of Nrf2 against CIN.

CIN is the third most common cause of hospital-acquired acute renal injury and is associated with a longer hospital stay and increased mortality^4,17,18^. Several therapeutic strategies have been studied for the treatment and prevention of CIN. However, none have been confirmed to be consistently beneficial for the treatment of CIN, and their utility for CIN prevention remains controversial^19^. Even saline hydration, which is the only proven method for preventing CIN, is not always successful in protecting against renal function deterioration following CM administration^20,21^.

The pathophysiology of CIN has not been fully elucidated, although various mechanisms, such as the direct toxicity of the CM, hypoxic injury due to reduced renal blood flow, and oxidative stress, have been studied^22^. In particular, oxidative stress has been suggested to be closely associated with CIN^23^. However, inconsistencies in the protective effects of antioxidant treatment against CIN have highlighted the necessity of a targeted treatment based on its detailed pathophysiology^24,25^.

Nrf2 is a well-known transcription factor involved in the regulation of various antioxidant-related genes^26^. When cells are exposed to electrophiles or oxidative stress, Nrf2 is released from Keap-1, a negative regulator of Nrf2 serving a substrate adaptor protein for the E3 ubiquitin ligase complex; Nrf2 is then translocated to the nucleus, resulting in the transcriptional activation of targeted antioxidant genes^27–29^. Several studies have demonstrated that Nrf2 is associated with various kidney diseases. For example, the loss of Nrf2 increased proinflammatory mediators and renal vascular permeability in a mouse ischemia-reperfusion model^30^. Additionally, Nrf2 activation in T cells provided significant protection against ischemia-reperfusion-induced AKI^31^. Keap1 hypomorphic mice showed a significant increase in the expression of Nrf2, and a renoprotective effect was found in a unilateral ureteral obstruction model^32^. In studies using human kidney-2 cells and rats, sulforaphane, a Keap1 cysteine modulator that acts as an antioxidant by inhibiting the Keap1-Cul3 complex function of ubiquitinating Nrf2^33^, which promotes the translocation of Nrf2 to the nucleus, effectively mitigated ischemia-reperfusion or cisplatin-related renal dysfunction^34,35^. In addition, lupus-like autoimmune nephritis was observed in Nrf2-deficient female mice^36^.

The present study revealed the important role of Nrf2 in CIN. In *in vitro* and *in vivo* murine models of CIN, increased Nrf2 expression was manifested in tubular cells and kidneys following iohexol treatment, and this increase in expression was correlated with the degree of renal injury at different time points. Moreover, the loss of Nrf2 expression in mice exacerbated renal dysfunction and histologic tubular injury. Nrf2 inhibition also resulted in a decrease in tubular cell viability. These results support the canonical role of Nrf2 in CIN.

The loss of Nrf2 function has been associated with the accentuation of apoptosis in CIN. For example, CM induced renal tubular epithelial cell apoptosis, which was considered to be part of the mechanism of CIN^37^. In rat and mouse models of ischemia-reperfusion renal injury, an increase in Nrf2 activity in renal tubular cells mitigated apoptosis after renal injury^38,39^. Overall, the interplay between increased apoptosis and ROS production has been suggested in the pathogenesis of CIN^40,41^, and Nrf2 may have a key role in CIN by modulating both apoptosis and ROS production.

In this study, we tried to clarify the relationship between the Nrf2 pathway and CIN, and we focused on HO-1, one of the antioxidant enzymes associated with Nrf2. HO-1 expression was stimulated to counteract iohexol treatment-induced oxidative stress. Nrf2 inhibition hindered the activation of HO-1, resulting in an increase in ROS production and the exacerbation of CIN. HO-1 has been studied for its cytoprotective role in cisplatin nephrotoxicity, rapamycin-induced AKI, ischemia- and glycerol-induced AKI, and nephrotoxic serum nephritis^42–45^. In a previous study, HO-1 induction in rats prevented the increase in superoxide observed in the kidneys after contrast injection and attenuated the subsequent tubular cell apoptosis and renal dysfunction^23^. These findings suggest that controlling the expression of Nrf2 and antioxidant enzymes, such as HO-1, may be useful targets for preventing or treating CIN. The protective effect of the Nrf2 activator CDDO-Me against tubular cell injury after iohexol treatment supported the role of Nrf2 as a therapeutic target in CIN. The analogues of CDDO, CDDO-Me and CDDO-Im, are potent activators of the Nrf2 pathway^46,47^. CDDO-Me showed marked protective effects in the ischemic AKI model in mice and improved inflammation in CKD patients with type 2 diabetes^10,48^. CDDO-Im also prevented tubular damage progression in early phase renal ischemia-perfusion injury and cisplatin-induced nephrotoxicity in mice^49,50^. Recently, there was an attempt to utilize CDDO as a therapeutic strategy in humans; a class III trial with CDDO-Me was initiated for patients with diabetic nephropathy^51^. However, it was not successful because it had to be terminated early due to a higher rate of cardiovascular events in the CDDO-Me-treated group^51–53^. Although the exact causes and mechanisms of this phenomenon have not yet been clarified, further studies should examine the role of Nrf2 activators as a therapeutic option for renal pathologies, including CIN, and establish safe and appropriate therapeutic indications for kidney disease^54,55^.

This study has several limitations. First, the sample size of eight mice in each control group (WTcon and KOcon) might not be sufficient to detect a difference in biochemical markers. Second, the CIN model we adopted from previous studies required pretreatment with a nitric oxide synthase inhibitor and a prostaglandin synthesis inhibitor before iohexol injection^56,57^, and these treatments may cause physiological changes or additional insults, which could prevent our model from reflecting actual clinical situations.

In conclusion, we demonstrated the role of Nrf2 as a key factor mediating oxidative stress and apoptosis in CIN. We believe that studies targeting the activation of Nrf2 and its downstream pathway can facilitate the development of a strategy for the prevention and treatment of CIN; thus, future studies are warranted.

## Materials and Methods

### Animals and experimental design

Breeding pairs of Nrf2^+*/−*^ (B6.129 × 1-Nfe2 l2tm1Ywk/J) mice were purchased from Orient-Bio (Gyeonggi-do, Korea). Nrf2^+/+^ (wild type, WT) and Nrf2^−/−^ (knockout, KO) mice were generated after breeding, and genotyping was confirmed by PCR. All mice were housed in an animal facility with an alternating 12-h light/dark cycle at 20 °C and 60% relative humidity and acclimated for at least 7 days before experimentation. The mice were fed commercial chow and allowed free access to water until 12 h before the experiments. Because severe dehydration could result in significant differences in contrast injury between groups, the effect of dehydration in WT and KO mice was compared after 24 h of water deprivation. We found no significant differences in serum creatinine levels between WT and KO mice after 24 h of water deprivation (Supplementary Figure S3). Mice were assigned to one of four groups: (1) saline-treated wild-type control (WTcon; n = 8), (2) saline-treated Nrf2-knockout (KO) (KOcon; n = 8), (3) contrast medium (CM)-treated wild-type (WT_CM; n = 14), and (4) CM-treated KO (KO_CM n = 14). Iohexol (Omnipaque, 350 mg iodine/mL, 5 g iodine/kg; GE Healthcare, Princeton, NJ, USA) was used as the iodinated radiographic contrast agent in this study. CIN was induced in the mice via combination injection of a nitric oxide synthase inhibitor (NG-nitro-l-arginine methyl ester, 10 mg/kg; Sigma-Aldrich, St. Louis, MO, USA) and a prostaglandin synthesis inhibitor (indomethacin, 10 mg/kg; Sigma-Aldrich) as described previously^56,57^. All groups received NG-nitro-l-arginine methyl ester and indomethacin 30 min before each saline or iohexol injection, and all drugs were injected intraperitoneally. The experimental protocol was approved by the Institutional Animal Care and Use Committee of the Korea University College of Medicine, and the experiments were performed in accordance with the institutional animal care guidelines. All mice were anesthetized via intraperitoneal injection of ketamine and sacrificed 24 h after drug administration.

### Quantification of functional and morphological CIN upon nuclear factor erythroid-2-related factor 2 inhibition

Blood samples were obtained from the inferior vena cava, and both kidneys were harvested after full exsanguination. Serum creatinine concentrations were measured using a DetectX Serum Creatinine Kit (Arbor Assays, Ann Arbor, MI, USA). Kidneys were harvested, washed with saline, fixed with 4% paraformaldehyde, and embedded in paraffin. Renal sections were stained with periodic acid-Schiff (PAS) stain, and CM-related injury, particularly in the proximal tubules and outer medulla, was examined. Ten randomly chosen, non-overlapping fields from the same section of kidney were captured (×400, Olympus BX50, Japan). The injured tubules were traced with the exclusion of Bowman’s capsule, peritubular capillaries and large vessels on each photograph using Image-Pro Plus (Media Cybernetics, Silver Spring, MD, USA). The injured area was expressed as the proportion of the traced field compared to the entire section.

### Nrf2 inhibition by small interfering RNA transfection in cultured cells

The rodent renal tubular cell line NRK-52E was obtained from the American Type Culture Collection (Manassas, VA, USA). Cells were cultured in Dulbecco’s modified Eagle’s medium (GIBCO, Life Technologies, Seoul, Korea), and the medium was replaced every 2 to 3 days. To assess the role of Nrf2 in CIN, Nrf2 expression in tubular cells was suppressed by transfection with a small interfering (si)RNA targeting Nrf2 (Santa Cruz Biotechnology, Santa Cruz, CA, USA). siRNA with complexed transfection agents (G-Fectin; Genolution, Seoul, Korea) was used as a negative control. A previously described *in vitro* model of radiocontrast nephropathy was adopted in the present study^56,58^. Approximately 24 h after transfection, the cells were exposed to iohexol (100 mg/mL) for 3 h or to an equal volume of saline as a control.

### Cell cytotoxicity assay after iohexol treatment and nuclear factor erythroid-2-related factor 2 inhibition

Cell viability, which reflected the degree of toxicity of iohexol, was measured using 3-(4,5-dimethyl-2-thiazolyl)-2,5-diphenyltetrazolium bromide (MTT) cytotoxic assays after treatment with saline (Cont) or iohexol (CM) and with or without Nrf2 inhibition (siRNA or CM + siRNA, respectively). The cells were incubated with 1 mg/mL MTT in sterile phosphate-buffered saline for 1 h at 37 °C, after which the resulting formazan crystals were dissolved in dimethyl sulfoxide. The optical density was quantified at 570 nm using a Beckman spectrophotometer (Brea, CA, USA).

### Immunohistochemistry

After deparaffinization, the kidney sections were hydrated in graded ethanol solutions, treated with 0.1% trypsin (Zymed, San Francisco, CA, USA) and 0.3% H_2_O_2_, and incubated with blocking serum (Vector Laboratories, Peterborough, UK) to prevent nonspecific detection. Then, the slides were incubated overnight at 4 °C with NGAL (1:1000; Abcam, Cambridge, UK) and F4/80 antibodies (1:100; AbD Serotec, Kidlington, UK), followed by incubation with biotin-conjugated secondary antibodies. For colorization, an avidin-biotin horseradish peroxidase complex and 3,3-diaminobenzidine substrate solution (Vector Laboratories, Burlingame, CA, USA) were applied to the slides at room temperature, and the slides were counterstained with hematoxylin (Sigma-Aldrich). Negative control slides were prepared by staining under identical conditions, with rabbit serum as a substitute for the primary antibody. All sections were examined in a blinded manner under a light microscope (Olympus BX-50; Olympus Optical, Tokyo, Japan). Finally, eight to ten high-power fields (HPFs) were captured (200× magnification), and the positively stained area for NGAL and the mean number of cells positive for F4/80 staining were calculated.

Apoptotic cells in the kidney were detected in paraffin-embedded kidney tissue sections with ApopTag Plus (Intergen, Purchase, NY, USA) according to the manufacturer’s protocol. Eight to ten HPFs were captured under 200× magnification, from which terminal deoxynucleotidyl transferase-mediated dUTP nick-end labeling (TUNEL)-positive cells were counted in the outer medulla and cortex; the mean number of positive cells was then calculated.

### Determination of reactive oxygen species and oxidative stress

Lipid peroxidation as an index of oxidative stress was assessed by malondialdehyde (MDA) production with the thiobarbituric acid reactive substance test (OxiSelect MDA Adduct ELISA Kit; Cell Biolabs Inc., CA, USA). ROS production was assessed directly in the *in vitro* experiment using fluorescence confocal microscopy. Cells were incubated with 10 μM dichlorofluorescein diacetate (DCF-DA; Invitrogen, Carlsbad, CA, USA) for 45 min at 37 °C, and the nuclei were stained with DAPI (Invitrogen). The cells were then examined using a Zeiss LSM confocal microscope with Zen2009 software (Carl Zeiss, Oberkochen, Germany; 2,000× magnification) and oil immersion with the appropriate filter sets for each dye. Quantitative ROS production in the *in vitro* experiment was measured at 530 nm absorbance using a Beckman spectrophotometer.

### Immunoblot analysis

Protein was extracted from tissues, and protein concentrations were determined using Bradford reagent (Bio-Rad, Hercules, CA, USA). The cytosolic fractions of kidney tissues were separated for cytochrome c measurement after the nuclear and mitochondrial fractions were isolated using an NE-PER Nuclear and Cytoplasmic Extraction Kit (Thermo Scientific, Waltham, MA, USA) and Mitochondria Isolation Kit (Thermo Scientific) according to the manufacturer’s protocols. Next, 100 µg of protein was separated by sodium dodecyl sulfate-polyacrylamide gel electrophoresis under denaturing conditions and electroblotted onto polyvinylidene fluoride membranes (pore size: 0.45 μm; Millipore, Bedford, MA, USA). The membranes were incubated with 5% nonfat dry milk in Tris-buffered saline-Tween-20 buffer for 1 h at room temperature and then hybridized with polyclonal antibodies against Nrf2 (1:1,000; Abcam, Cambridge, UK), heme oxygenase-1 (HO-1, 1:1,000; Enzo Life Sciences, NY, USA), cytochrome c (1:1000; BD Biosciences, CA, USA), and caspase-3 (1:1,000; Cell Signaling Technology, Danvers, MA, USA) overnight at 4 °C. The antibody specificity of Nrf2 was tested with KO mice (Supplementary Figure S4). Finally, the membranes were reacted with a horseradish peroxidase-conjugated secondary antibody (1:2,000; Vector Laboratories) for 90 min at room temperature. Signals were visualized by chemiluminescent detection according to the manufacturer’s protocol (Amersham Pharmacia Biotech, London, UK), and the signal densities, measured using ImageJ (NIH, Bethesda, MD, USA), were compared among the treatment groups. Equal protein loading was confirmed using an anti-β-actin antibody (1:20,000; Santa Cruz Biotechnology) and anti-lamin B1 antibody (1:1,000; Santa Cruz Biotechnology).

### Cell proliferation after Nrf2 activator treatment

NRK-52E cells were treated with the Nrf2 activator bardoxolone methyl (CDDO-Me; Toronto Research Chemicals, Toronto, ON, Canada) to confirm the role of Nrf2 in iohexol-induced tubular injury. Approximately 1 h after treatment with 5 nM CDDO-Me, cells were treated with saline or iohexol for 3 h with or without Nrf2 siRNA transfection, and cell viability was measured using 5-(3-carboxymethoxyphenyl)-2H-tetrazolium inner salt (MTS) cell viability and proliferation assays (Promega Corp., Madison, WI, USA) according to the manufacturer’s protocol. The optical density was measured at 490 nm using a Beckman spectrophotometer.

### Statistical analysis

The data are expressed as the means ± standard error. Differences among groups were compared by analysis of variance with Bonferroni correction using SPSS software (ver. 20.0; IBM Corp., Armonk, NY, USA). Differences with *p* values < 0.05 were considered to be significant.

## Supplementary information

Dataset 1
