# Supplementary material for: The role of nuclear factor erythroid-2-related factor 2 expression in radiocontrast-induced nephropathy
Source: Sci Rep. 2019 Feb 22;9:2608. doi: 10.1038/s41598-019-39534-2 (PMC6384919; doi:10.1038/s41598-019-39534-2)
Supplement: Supplementary file 1 — Dataset 1 [file 41598_2019_39534_MOESM1_ESM.pdf]

# The role of nuclear factor erythroid-2-related factor 2 expression in radiocontrast-induced nephropathy

Ji Eun Kim, M.D[a], So Yeon Bae[b], Shin Young Ahn, M.D., PhD.[a], Young Joo Kwon M.D.,PhD.[a], and Gang Jee Ko M.D.,PhD.[a]

**Department of Internal Medicine, Korea University College of Medicine, Seoul, Korea[a]**

**Nephrology Research Institution, Korea University Guro Hospital, Seoul, Korea[b]**

*** Corresponding Author:** Gang Jee Ko, MD, PhD

Division of Nephrology, Department of Internal Medicine,

Korea University Guro Hospital, Korea University College of Medicine,

#148, Gurodongro, Gurogu, Seoul, 152-703, Korea

Tel: +82-2-2626-3039, Fax: +82-2-2626-1798,

E-mail: lovesba@korea.ac.kr

## Supplementary Figures.

### Supplemental Figure S1

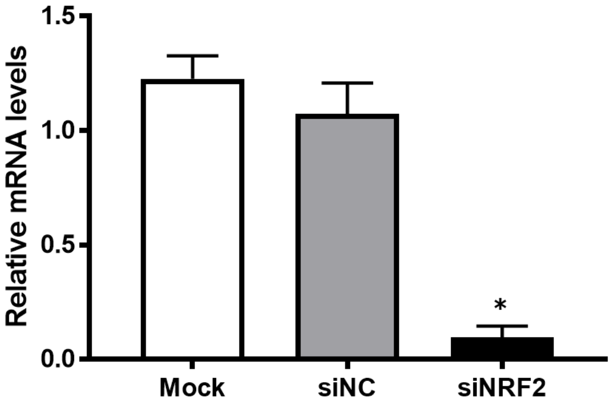


**Supplementary Figure S1.** Efficacy of siRNA on inhibition of Nrf2 expression

Confirmation of inhibitory efficacy of siRNA by comparison of Nrf2 expression. The siRNA targeting Nrf2 transfection group showed significant decrease of Nrf2 expression compared with other controls. *$p < 0.05$ vs. Mock and siNC

Abbreviation: Mock, control group treated with transfection reagents only; siNC, control group treated with scrambled stealth siRNA; siNRF2, siRNA targeting Nrf2 transfection group.

**Supplemental Figure S2**

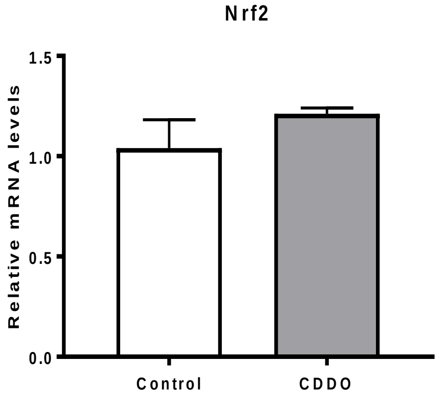


**Supplementary Figure S2.** Nrf2 expression increases after CDDO-Me treatment.

Treatment of CDDO-Me on NRK-52E cells increased Nrf2 expression compared to control group which only treated with DMSO solution (p=0.05).

**Supplemental Figure S3**

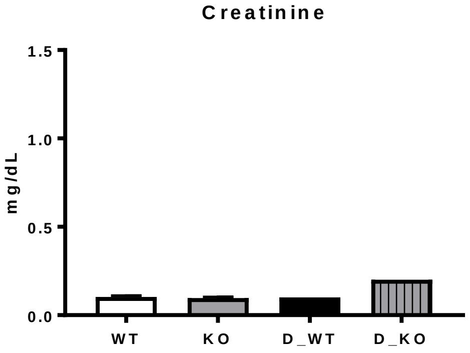


**Supplementary Figure S3.** Changes in creatinine in KO and control mice before and after dehydration

The changes in creatinine were not significant after dehydration for 24 hours in both KO and control mice.

Abbreviation: WT, wild type mice; KO, knockout mice; D_WT, wild type mice after dehydration; D_KO, knockout mice after dehydration.

**Supplemental Figure S4**

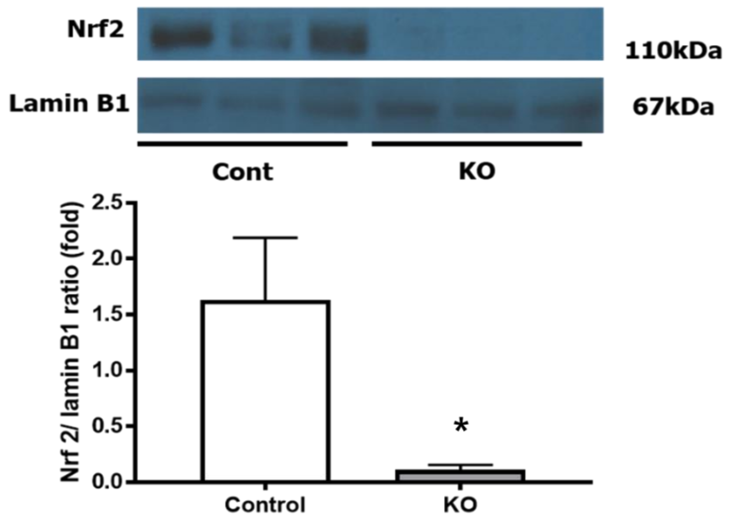


**Supplementary Figure S4.** Comparison of Nrf2 expression in control and KO mice for confirming specificity of Nrf2 antibody.

The Nrf2 expression in nucleus was barely observed in KO mice compared to wild type control in western blot analysis. The cropped gels are used in the figure, and full-length gels are presented in Supplementary Figure S12. *$p<0.05$ vs. Control and KO

## Supplemental Figure S5

Nrf2

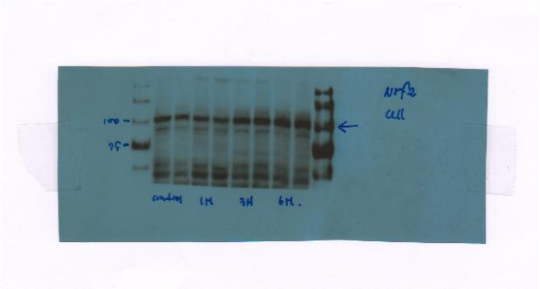


β-actin

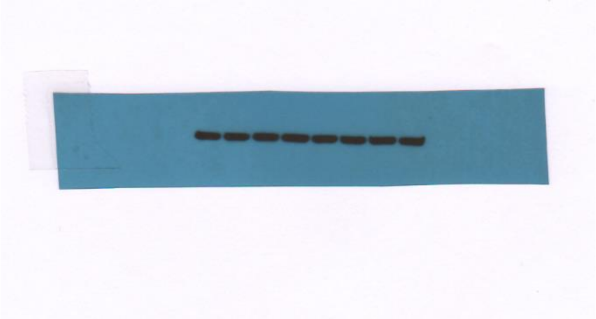

**Supplementary Figure S5.** Full length blots of Figure 1A. Brightness was adjusted during processing these gels

**Supplemental Figure S6**

cleaved caspase 3

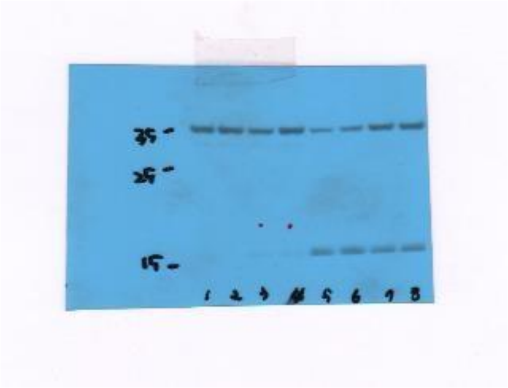


β-actin

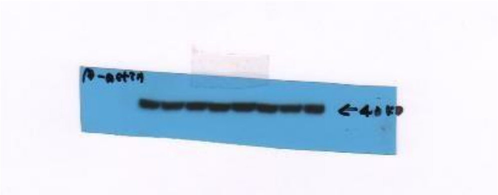

**Supplementary Figure S6.** Full length blots of Figure 1B. Brightness was adjusted during processing these gels

**Supplemental Figure S7**

Nrf2

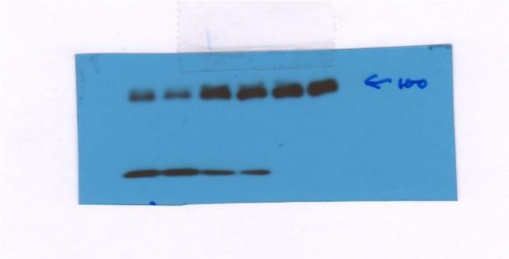


β-actin

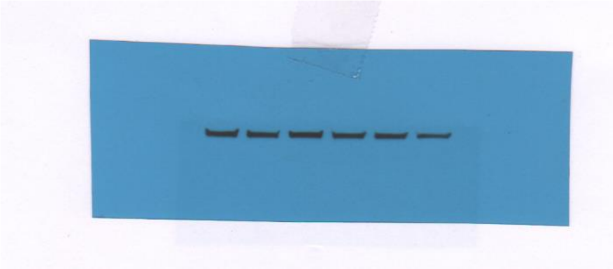

**Supplementary Figure S7.** Full length blots of Figure 1C. Brightness was adjusted during processing these gels

**Supplemental Figure S8**

cleaved caspase 3

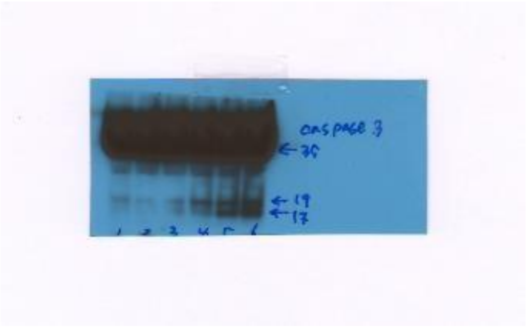


β-actin

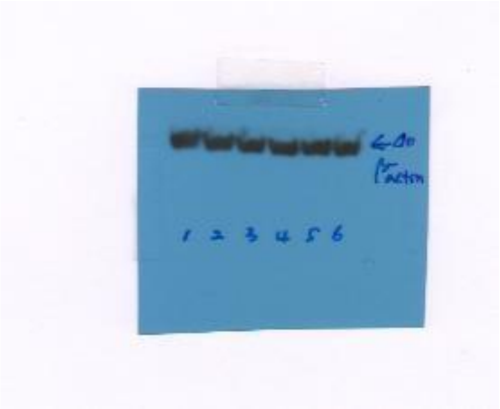


**Supplementary Figure S8.** Full length blots of Figure 1D. Brightness was adjusted during processing these gels

## Supplemental Figure S9

Cleaved caspase 3

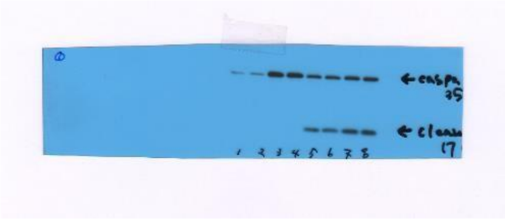


β-actin

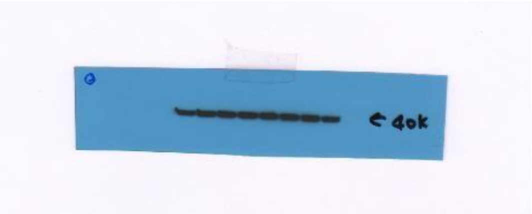


**Supplementary Figure S9.** Full length blots of Figure 4. Brightness was adjusted during processing these gels

**Supplemental Figure S10**

(Left) HO-1

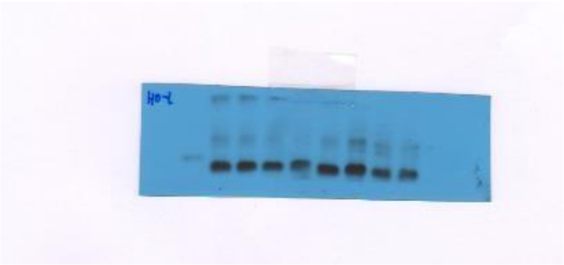


(Left) β-actin

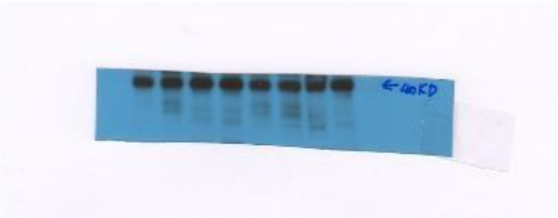


(Right) HO-1

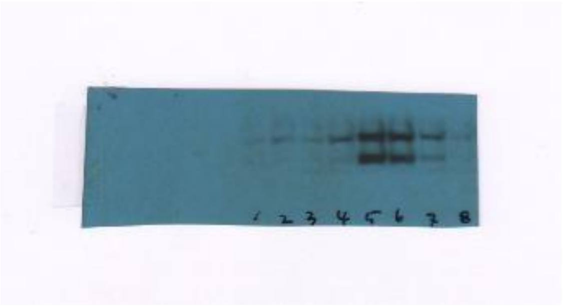


(Right) β-actin

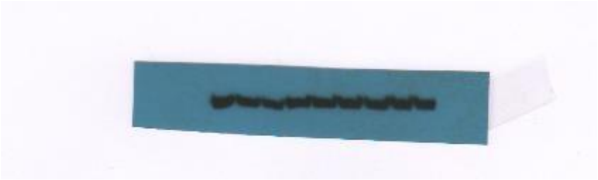

**Supplementary Figure S10.** Full length blots of Figure 6A. Brightness was adjusted during processing these gels

## Supplemental Figure S11

(Left) cytochrome C

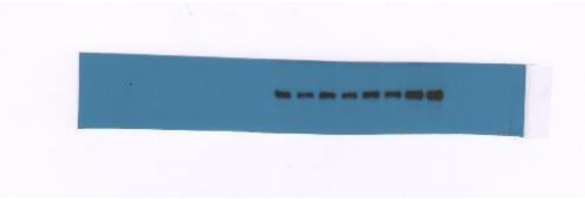

(Left) β-actin

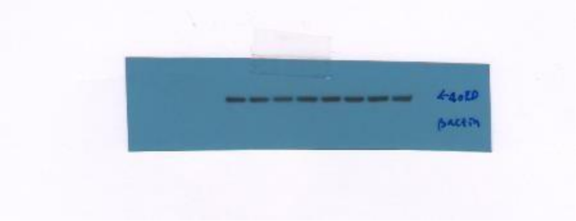

(Right) cytochrome C

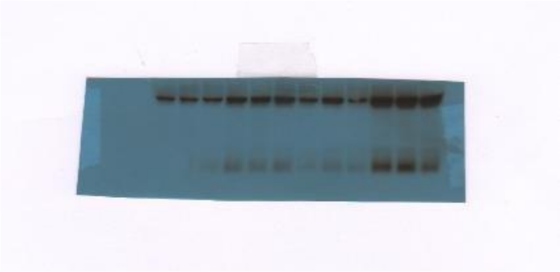

(Right) β-actin

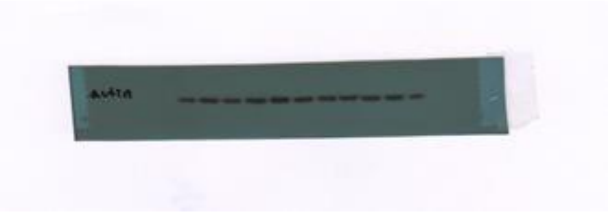

**Supplementary Figure S11.** Full length blots of Figure 6B. Brightness was adjusted during processing these gels

**Supplemental Figure S12**

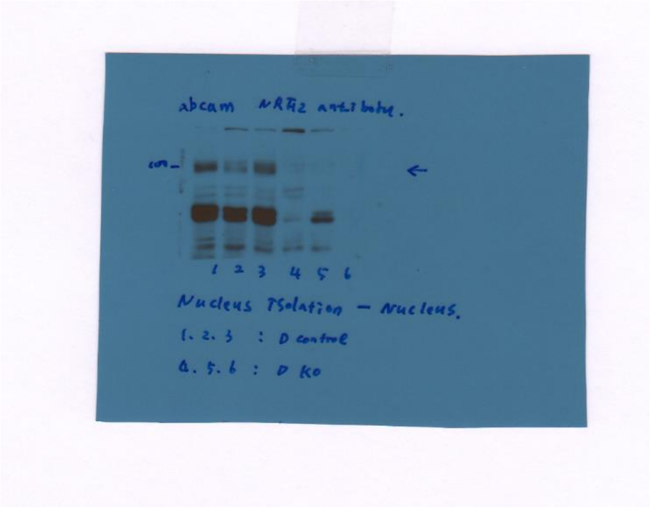


**Supplementary Figure S12.** Full length blots of Supplemental Figure S4. Brightness was adjusted during processing these gels
